# Concerns about using a digital mask to safeguard patient privacy

**DOI:** 10.1038/s41591-023-02439-9

**Published:** 2023-07-18

**Authors:** Matthieu Meeus, Shubham Jain, Yves-Alexandre de Montjoye

**Affiliations:** Department of Computing, Imperial College London, London, UK

**Keywords:** Scientific community, Research data

arising from 10.1038/s41591-022-01966-1 (2022)

Sharing data is crucial for advancing medical research but should not come at the expense of patient privacy. Yang et al.^1^ proposed to apply a digital mask (DM) to a facial image with the goal of retaining information relevant for medical diagnosis while ‘irreversibly erasing identifiable features’, making the data ‘anonymous’^2^. The masking approach consists of a three-dimensional reconstruction from a two-dimensional facial image, to be rendered back as the DM. The paper shows that diagnosis of ocular conditions using masked reconstructions of facial videos is both accurate and consistent with the diagnosis on original (unmasked) videos. The authors show that the DM can evade AI-powered facial recognition systems, which underpins their claim that the method preserves privacy.

Although sharing data for medical diagnosis while preserving privacy is an important line of research, we believe the evaluation setup in Yang et al. to be inadequate, raising serious questions with regard to the risk to patient privacy posed by the proposed masking method. The facial recognition setup used by the authors as validation of the privacy-preserving capabilities of the DM assumes that an attacker attempting to identify a patient will try to match a mask to a database of faces (a Mask2Face approach) using a facial recognition algorithm. We argue that this setup and the corresponding empirical results reported by the authors do not properly evaluate the risk of reidentification. Indeed, a simple change to the setup, assuming the masking algorithm is available, allows an attacker to mask the faces before running a facial recognition algorithm on the now more comparable database of masked faces (a Mask2Mask approach).

The code made available by the authors is not sufficient to apply their masking technique to an image nor to evaluate the risk of reidentification. Similarly, the data they used to evaluate the preserving capabilities of their method are not available. To evaluate the risk of reidentification posed by the Mask2Mask approach, we instead used a similar linear face reconstruction model called FLAME^3^, more specifically the RingNet implementation^4^, to produce the facial masks. To evaluate the risk of reidentification, we used the Insightface implementation of the ArcFace^5,6^ facial recognition model adopted by Yang et al. Finally, we used the YouTube Faces Database^7^ as a dataset (Supplementary Information).

In this comparable setup, we first replicated the reidentification results obtained by Yang et al. We randomly sampled two frames from facial videos for each individual; then, we used one image in its original state as a reference image in the database, while the other image was used to compute the mask on a black background as the query image to be matched against the database (Mask2Face). Figure 1 shows that we obtained a rank-1 accuracy, the percentage of the time the algorithm identifies the right person in the database—the metric used by the authors for the risk of reidentification, of 0.7%, a value very similar to the 0.5% reported by Yang et al.

**Fig. 1:**
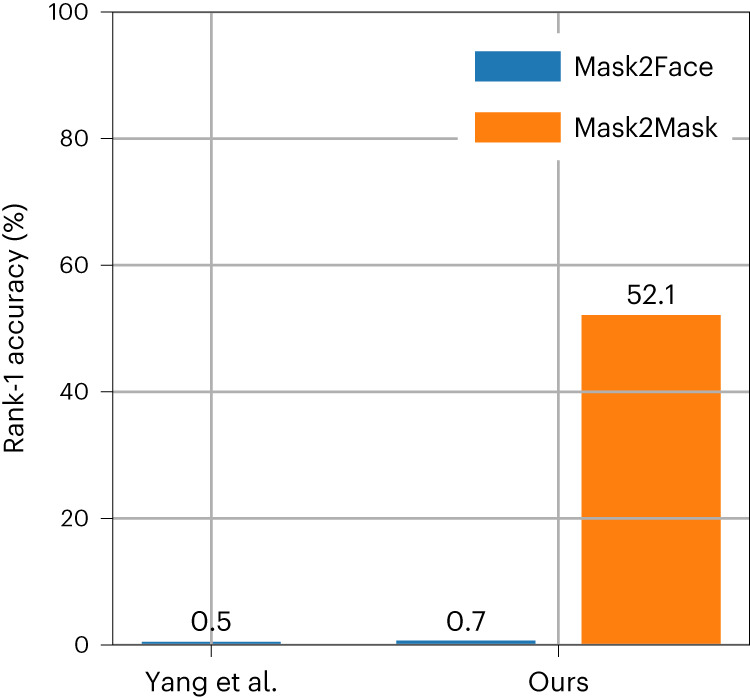
Mask2Mask achieves a reidentification accuracy of 52%. Rank-1 accuracy in facial recognition across 2 methods: (1) ‘Mask2Face’: mask as query image and original image as database image; (2) ‘Mask2Mask’: mask as query image and mask as database image. Results for Yang et al. ^1^ are based on analysis of 405 individuals, while results for our analysis (‘ours’) are based on analysis of 555 individuals.

We then modified the setup to evaluate the risk posed by the Mask2Mask approach. In this setup, an attacker would obtain a rank-1 accuracy of 52% (Fig. 1) meaning that they can now correctly reidentify an individual more than half the time, an increase of 100-fold over the results reported by Yang et al. for the risk of reidentification (0.5%).

These results are furthermore only a lower bound on the actual risk. First, we used only the reconstructed face to reidentify patients in the protected database. The proposed method releases not only the reconstructed face but also the reconstructed eyeballs and eyelids. These are likely to provide further information to an attacker aiming to reidentify patients. Second, both our and the authors’ reidentification results stem from readily available facial recognition algorithms. These are trained to identify individuals in pictures, based on detected facial patterns, but are not optimized for DM-reconstructed images. It is likely that better reidentification algorithms could be developed to reidentify masked patients^8,9^. An attacker leveraging the additional information available, such as eyeballs, and better reidentification algorithms is thus likely to be able to reidentify an individual with an even higher rank-1 accuracy than the one we report here.

Contrary to Yang et al.’s claims, our results show that the DM does not irreversibly erase identifiable features of a facial image. Anonymization requires, from both technical and legal perspectives, much more than an individual not being recognized by the human eye. Rather, GDPR Recital 26 (ref. ^10^) requires all means that are reasonably likely to be used by an attacker to be considered, and China’s Personal Information Protection Law requires ‘mak[ing] it impossible to distinguish specific natural persons and impossible to restore’^11^. Similarly, patients’ privacy cannot, in general, be considered protected if it relies on an algorithm being kept secret now and forever^12^. In the case of the DM, the algorithm is published, relies on existing methods and is proposed to be deployed broadly.

Sharing data for research, in particular medical research, is highly beneficial to the scientific community and beyond, but cannot come at the expense of patient privacy and, ultimately, trust. While we appreciate the aims of Yang et al. to enable privacy-preserving patient diagnosis, ad hoc and inadequately tested methods have damaged patient trust before and put access to data for research at risk^13^. Although methods providing formal privacy guarantees are preferred, they are not always within reach or free from implementation issues. Any anonymization methods proposed therefore need to be extensively, and if possible adversarially, tested to ensure that privacy is preserved before data is shared.

## Reporting summary

Further information on research design is available in the Nature Portfolio Reporting Summary linked to this article.

## Online content

Any methods, additional references, Nature Portfolio reporting summaries, source data, extended data, supplementary information, acknowledgements, peer review information; details of author contributions and competing interests; and statements of data and code availability are available at 10.1038/s41591-023-02439-9.

## Supplementary information

Supplementary Information Supplementary Methods

Reporting Summary

## Data Availability

The YouTube Faces Database is a publicly available dataset; for access, refer to ref. ^7^. The code and/or instructions for the replication of Yang et al.’s results as well as ours are available at https://github.com/computationalprivacy/unmask/.
