# Supplementary material for: Concerns about using a digital mask to safeguard patient privacy
Source: Nat Med. 2023 Jul 18;29(7):1658–9. doi: 10.1038/s41591-023-02439-9 (PMC10353923; doi:10.1038/s41591-023-02439-9)
Supplement: Supplementary file 1 — Supplementary Methods [file 41591_2023_2439_MOESM1_ESM.pdf]

nature medicine

Matters arising

https://doi.org/10.1038/s41591-023-02439-9

Concerns about using a digital mask to safeguard patient privacy

In the format provided by the authors and unedited

Supplementary Information

Masking algorithm

As an alternative for the FaceWarehouse model [1] used in Yang et al., the authors suggested using the linear FLAME model for facial mesh reconstruction. We agree that these models are comparable, as they both offer a linear paradigm for face reconstruction -singular value decomposition in FaceWarehouse and principal component breakdown in FLAME-computed on their respective datasets.

In order to determine the weights for the FaceWarehouse model, Yang et al. implemented an optimization scheme to minimize landmark projection errors between the original image and the three-dimensional reconstruction. For FLAME, RingNet provides a convolutional encoder as an alternative to the landmark detection and optimization, enabling us to generate the linear 3D FLAME reconstruction directly from a 2D image. Hence, we use the RingNet pipeline to generate all 3D facial meshes for the preprocessed images.

Finally, we use the RingNet rendering process and a fine-tuned landmark detection model - that includes landmarks for the forehead- [2] to render the 3D facial mesh and cut out the 2D mask.

Data collection and processing
In order to accurately reproduce the setup of the authors, we used facial images from the YouTube Faces Database, which contains 3,425 videos of 1,595 individuals. This dataset allows us to randomly sample frames from the same video for each individual, the setup used by Yang et al.

The masking algorithm requires a reasonable facial image of one individual as input. To render our dataset as similar as possible to the one used by Yang et al., videos captured in laboratory conditions for medical diagnosis, we apply the following filtering mechanism. We use the RetinaFace model for face detection and only consider images where exactly one face is detected. For the selected images we leverage FacePosePytorch [3] to estimate the face pitch, yaw and roll based on detected landmarks by a pre-trained PFLD model [4]. We only consider faces where the estimated pitch and yaw are below 20°. If a frame meets the requirements, we crop it to 1.5x the face bounding box.

After filtering the frames, sampling two frames per video per individual and applying the RingNet masking pipeline to all frames, we end up with 555 unique individuals as input for our facial recognition, which makes our setup comparable to the one used in Yang et al. (405 individuals).

Facial Recognition
In line with Yang et al. we use ArcFace as the facial recognition algorithm. Specifically, we use the InsightFace implementation of the RetinaFace model for face detection and the ArcFace model trained on WebFace600K [5] for face recognition. We use the model to compute embeddings for both the query as well as database images and use cosine similarity to determine the distance between two images. Given a query image, the predicted identity corresponds to the database image whose embedding is the closest to the embedding of the query image. As a metric, we use rank-1 accuracy for all 555 individuals which corresponds to the percentage of the time the algorithm identifies the right person in the database.

References

[1] Cao, C., Weng, Y., Zhou, S., Tong, Y., & Zhou, K. (2013). Facewarehouse: A 3d facial expression database for visual computing. *IEEE Transactions on Visualization and Computer Graphics*, *20*(3), 413-425.

[2] 81 Facial Landmarks Shape Predictor. https://github.com/codeniko/shape_predictor_81_face_landmarks [Accessed November 1st, 2022]

[3] FacePose_Pytorch. https://github.com/WIKI2020/FacePose_pytorch [Accessed November 1st, 2022]

[4] Guo, X., Li, S., Yu, J., Zhang, J., Ma, J., Ma, L., ... & Ling, H. (2019). PFLD: A practical facial landmark detector. *arXiv preprint arXiv:1902.10859*.

[5] Zhu, Z., Huang, G., Deng, J., Ye, Y., Huang, J., Chen, X., ... & Zhou, J. (2022). WebFace260M: A Benchmark for Million-Scale Deep Face Recognition. *IEEE Transactions on Pattern Analysis and Machine Intelligence*.
